# Characteristics and survival outcomes related to the infra-pyloric lymph node status of gastric cancer patients

**DOI:** 10.1186/s12957-018-1412-8

**Published:** 2018-06-20

**Authors:** Wei-Han Zhang, Xiao-Hai Song, Xin-Zu Chen, Kun Yang, Kai Liu, Zhi-Xin Chen, Zong-Guang Zhou, Jian-Kun Hu

**Affiliations:** 1 Department of Gastrointestinal Surgery and Laboratory of Gastric Cancer, State Key Laboratory of Biotherapy, West China Hospital, Sichuan University, and Collaborative Innovation Center for Biotherapy, No. 37 Guo Xue Xiang Street, Chengdu, Sichuan Province China; 2 Department of Gastrointestinal Surgery and Laboratory of Digestive Surgery, State Key Laboratory of Biotherapy, West China Hospital, Sichuan University, and Collaborative Innovation Center for Biotherapy, Chengdu, China

**Keywords:** Station no. 6, Lymphadenectomy, Gastric cancer, Prognosis

## Abstract

**Background:**

To study metastasis to the infra-pyloric (no. 6) lymph nodes and their subgroups and the related risk factors of gastric cancer patients.

**Methods:**

Gastric cancer patients who underwent gastrectomy with complete postoperative pathological information on the no. 6 lymph node station and its subgroups from January 1, 2008, to December 31, 2011, were included. The clinicopathological characteristics and survival outcomes were analyzed.

**Results:**

A total of 121 patients were included; they had 6.1 ± 7.7 positive lymph nodes, and 35.1 ± 14.2 lymph nodes were examined. The overall lymph node positivity rate was 67.8% (82/121) with a positivity rate of 28.1% (34/121) for the no. 6 lymph nodes. The metastasis rate was 6.6% for the no. 6a nodes, 6.6% for the no. 6b nodes, and 21.5% for the no. 6c nodes. Also, no. 8a (OR = 1.329, *p* = 0.017) and no. 9 (OR = 1.250, *p* = 0.022) nodal positivity and lower third tumor location (OR = 1.278, *p* = 0.001) were independent risk factors for no. 6 lymph nodal metastasis. There was a significant survival difference between patients with positive and negative no. 6 lymph nodes and patients with metastasis to other lymph node stations (*p* <  0.001).

**Conclusions:**

Patients with no. 6 lymph node metastasis have poor survival outcomes. Complete infra-pyloric lymphadenectomy is necessary and crucial for gastric cancer patients.

## Background

Gastric cancer is among the most common malignant diseases, particularly in East Asian countries, such as Japan, Korea, and China [1–3]. Surgical treatment combined with peri-operative chemotherapy is its primary surgical treatment strategy [4–6]. Of the surgical treatment principles of gastric cancer, the tenet is radical lymphadenectomy. The removal of potential metastases to perigastric lymph nodes can reduce the risk of tumor recurrence and extend survival outcomes for gastric cancer patients. Recently, standard D2 lymphadenectomy has been acknowledged as the universal surgical treatment standard for advanced gastric cancers [5–7]. However, the lower third of the stomach is the most common site of gastric cancer, which tends to have lymph node metastasis in the infra-pyloric area [8]. A previous study focused on patients with metastasis in single lymph node station and found out 36.5% lower third gastric cancer patients have no. 6 lymph node metastasis [9]. According to the Japanese Gastric Cancer Classification, lymph nodes located in the infra-pyloric area were categorized as the no. 6 station [10, 11], which is a significant lymphatic channel that drains the distal part of the stomach. Additionally, metastasis to no. 6 lymph nodes is quite common for gastric cancers, and complete resection of this station is a crucial surgical procedure [12–14].

Currently, Japanese studies presented a subgrouping method for the no. 6 station, which divided lymph nodes in the infra-pyloric area into the no. 6i, 6v, and 6a groups according to topographic anatomical considerations [15, 16]. Accordingly, the no. 6v group comprises the nodes that lie along the proximal part of the right gastroepiploic vein (RGEV), the no. 6a group comprises the nodes along the right gastroepiploic artery (REGA), and the no. 6i group comprises the nodes along the infra-pyloric artery (IPA). Moreover, based on the embryology of the infra-pyloric area and according to laparoscopic technology, Japanese investigators also focused on the surgical technique used during lymphadenectomy of the infra-pyloric area [16, 17].

We have recognized the importance of infra-pyloric lymphadenectomy for years. In the year 2011, our study group reported a method of sub-dividing the infra-pyloric lymph nodes [18]. The no. 6 lymph nodes were grouped into three subgroups: the left side of the confluence of the anterior superior pancreaticoduodenal vein (ASPDV) and the REGV (no. 6a), the right side of the confluence of the ASPDV and the REGV (no. 6b), and the root of the RGEA to the right side of its first branch to the stomach (no. 6c).

However, Chinese gastric cancer patients usually have an advanced tumor stage at initial diagnosis and a relatively high metastasis rate to the infra-pyloric lymph nodes (41.3% in our previous study) [18]. Therefore, a precise and thorough lymphadenectomy of the infra-pyloric area is critical. In the present study, we aim to evaluate the clinicopathological characteristics of lymph nodes in the infra-pyloric area and patient survival outcomes.

## Methods

### Ethical statement

This study was based on the information gathered from the database of the Surgical Gastric Cancer Patient Registry of West China Hospital (WCH-SGCPR) under registration number WCH-SGCPR-2017-02 [19]. The establishment of this database was approved by the Biomedical Ethical Committee of West China Hospital.

Besides, patients were not written inform consent because this is a retrospective study, but the individual information were anonymized prior to statistical analysis.

### Patients

Medical information on patients who underwent treatment with complete data on the no. 6 lymph nodes and their subgroups from January 1, 2008, to December 31, 2011, were collected from the database of the WCH-SRCPR [19]. The inclusion criteria were the following: (1) pathologically proven primary gastric adenocarcinoma, (2) patients with radical distal gastrectomy or total gastrectomy, (3) patients without preoperative chemotherapy or radiotherapy, and (4) complete information on infra-pyloric lymph nodes and their subgroups. The general clinicopathological characteristics of these patients, including age (years), gender (male or female), tumor location (upper, middle, and lower) and tumor size (cm), macroscopic type (type 0, I-IV), histologic grade (moderately or poorly differentiated), operation type (open or laparoscopic surgery), resection patterns (distal gastrectomy or total gastrectomy), operation time (min), blood loss (ml), and TNM stage and lymph nodal status in each group, among other features, were also retrieved for analysis in this study.

### The subgroups of the no. 6 lymph nodes

The no. 6 lymph nodes were divided into three subgroups according to the anatomic structure observed during the operation when the specimen was excised [18]. In addition to the separation of the no. 6 lymph nodes from the specimen, all of the other regional lymph nodes were immediately grouped and labeled by the surgeon according to the definitions of the Japanese classification [10]. Specifically, in the present study, the no. 6a station is located at the left side of the juncture of the RGEV and ASPDV; the no. 6b station is located to the right of the pancreatic anterior segments of the RGEV and ASPDV; and the no. 6c station is located from the root to the first branch of the RGEA and includes the nodes along superior pancreatic segment of the RGEV.

### Surgery and no. 6 lymphadenectomy

All the gastric cancer patients underwent surgical treatment in the Department of Gastrointestinal Surgery, West China Hospital, Sichuan University. The surgical treatment principles were based on the Japanese Gastric Cancer Treatment Guidelines [5, 10].

During the no. 6 lymphadenectomy, the stomach was retracted, and the gastrocolic omentum along the edge of the transverse colon was transected. Then, from the anatomical space between the anterior and posterior lobes of the transverse mesocolon, the right part of the anterior lobe to the inferior border of the pancreas was removed. Next were the key procedures, which are aimed to explore the right operative plane and achieve en bloc infra-pyloric lymphadenectomy. From the fused fascia of the right part of the transverse mesocolon and the pancreatic capsule to the side of descending part of the duodenum, remove the membrane on the surface of pancreatic head, providing subsequent exposure of the confluence of REGV and ASPDV. The root of the REGV was transected above the confluence and then upward to remove the no. 6a and no. 6b nodes. Continuing upward, the gastroduodenal artery was exposed, and the root of the REGA was transected. Finally, the inferior pyloric artery (IPA) was exposed and ligated, and the inferior wall of the duodenal bulb was skeletonized. Thus, the infra-pyloric lymph nodes were en bloc removed with the gastric specimen.

### Pathology

Pathological examinations were performed by the pathologists in the Department of Pathology, West China Hospital. Intraoperative frozen sections were performed to ensure negative margins. All specimens were fixed in 10% formaldehyde solution. The tumor staging was performed according to the Japanese Gastric Cancer Classification 3rd English Edition and 7th edition TNM classification of gastric cancer published by the American Joint Committee on Cancer (AJCC) [11, 20].

### Postoperative treatment and follow-up

Postoperative adjuvant chemotherapy was recommended for patients with advanced tumor stage or early tumor stage with positive lymph nodes. Combinations of fluoropyrimidine and platinum regimens every 3 weeks for six to eight cycles were the first-line treatment strategies.

Postoperative follow-up was performed at the postoperative outpatient visits. All patients were recommended to undergo follow-up every 3 to 6 months during the first 3 years and at least once yearly during the subsequent years. Follow-up information was also collected from the database and updated to January 1, 2018. Reasons for patients loss to postoperative follow-up were the refusal to attend the outpatient visits or loss of contact due to a change of telephone number and address. Ultimately, 116 patients had complete postoperative follow-up information with a 95.9% follow-up rate and a median follow-up duration of 89.6 (28–104) months.

### Statistical analyses

Statistical analyses and graphics were conducted using GraphPad Prism 6 and R software (version 3.1.2.). Spearman’s correlation test was used to analyze the correlation of the metastatic status among all lymph node groups. Univariate analyses and multivariate analyses were performed to identify risk factors for metastases to no. 6 lymph nodes by the logistic regression model and evaluated by odds ratios (ORs) and 95% confidence intervals (CIs). Survival outcomes were analyzed by the Kaplan–Meier method and compared by log-rank test. Variables were identified by univariate analysis, further examined by multivariate analysis, and measured by hazard ratios (HRs) and 95% CI. Two sides of *p* values < 0.05 were considered statistically significant in the present study.

## Results

### Clinicopathological characteristics

Medical information on 121 patients was collected in the present study. The general clinicopathological characteristics of these patients were presented in Table 1. Their mean age was 55.0 ± 12.5 years; 81 (66.9%) patients were male, and only 24.0% of them were confirmed as early stage tumor by the pathological examination.

**Table 1:** Clinicopathologic features of the included patients

Characteristics		*N* = 121 (%)
Age	Years, mean ± SD	55.0 ± 12.5
Gender	Male/female	81 (66.9)/40 (33.1)
Tumor Location	U/M/L	24 (19.8)/24 (19.8)/73 (60.3)
Tumor size	cm, mean ± SD	5.0 ± 2.9
Histologic grade	Moderate/poor	19 (15.7)/102 (84.3)
Macroscopic type	Type 0-II/type III-IV	84 (69.4)/37 (30.6)
Operation type	Open/laparoscopic	81 (66.9)/40 (33.1)
Resection patterns	DG/TG	66 (54.5)/55 (45.55)
Operation time	min, mean ± SD	248.5 ± 44.0
Blood loss	ml, mean ± SD	154.8 ± 97.9
T stage	T1/T2/T3/T4	29 (24.0)/12 (9.9)/7 (5.8)/73 (60.3)
N stage	N0/N1/N2/N3	39 (32.2)/22 (18.2)/13 (10.7)/47 (38.8)
M stage	M0/M1	110 (90.9)/11 (9.1)
TNM stage	I/II/III/IV	31 (25.6)/21 (17.4)/46 (38.0)/11 (9.1)
Chemotherapy	Yes/no	74 (61.2)/47 (38.8)
Positive lymph nodes	Numbers, mean ± SD	6.1 ± 7.7
Examined lymph nodes	Numbers, mean ± SD	35.1 ± 14.2

*U* upper, *M* middle, *L* lower, *DG* distal gastrectomy, *TG* total gastrectomy

### Metastasis status of and correlation analysis

Overall, the lymph node positivity rate was 67.8% (82/121) in these patients. The numbers of positive lymph nodes and examined lymph nodes were 6.1 ± 7.7 and 35.1 ± 14.2, respectively. The lesser curvature area and the celiac axis area were the most frequent metastatic regions (Table 2). In detail, 34 (28.1%) patients had metastasis to no. 6 lymph nodes, and the metastatic rates were 6.6% for no. 6a, 6.6% for no. 6b, and 21.5% for no. 6c stations.

**Table 2:** Metastatic status of D2 tier lymph nodes of included patients

Lymph node station	Positive cases, *n* (%)	Numbers of metastatic LNs Mean ± SD	Numbers of harvested LNs Mean ± SD
No. 1	74 (61.2)	0.3 ± 0.9	1.4 ± 1.7
No. 3	58 (47.9)	1.8 ± 2.5	5.4 ± 4.6
No. 4d	32 (26.4)	0.7 ± 1.5	4.4 ± 3.7
No. 4sb	3 (2.5)	0.0 ± 0.2	0.7 ± 1.4
No. 5	15 (12.4)	0.2 ± 0.5	0.7 ± 0.9
No. 6*	34 (28.1)	0.8 ± 1.6	5.0 ± 3.4
No. 6a	8 (6.6)	0.1 ± 0.7	0.9 ± 1.7
No. 6b	8 (6.6)	0.1 ± 0.4	0.9 ± 1.4
No. 6c	26 (21.5)	0.6 ± 1.4	3.2 ± 3.3
No. 7	40 (33.1)	0.7 ± 1.1	4.4 ± 3.2
No. 8a	21 (17.4)	0.2 ± 0.5	1.6 ± 1.2
No. 9	35 (28.9)	0.4 ± 0.9	2.9 ± 2.4
No. 11p	24 (19.8)	0.3 ± 0.8	2.2 ± 1.6
No. 12a	2 (1.7)	0.0 ± 0.1	0.4 ± 0.6

*There were 4 patients and 2 patients with two and four subgroup lymph node metastasis of no. 6 group

Correlation analyses were performed to identify the correlation of infra-pyloric lymph nodes and other lymph nodes. Among the three subgroups of infra-pyloric lymph nodes, the no. 6a and no. 6b subgroups (*r* = 0.20, *p* = 0.030) and the no. 6b and no. 6c subgroups (*r* = 0.35, *p* <  0.001) were significantly correlated. However, there was no significant correlation between the no. 6a and the no. 6c subgroups (*r* = 0.02, *p* = 0.804) (Table 3). In addition, no. 6a lymph nodal metastasis was significantly correlated with no. 3, 5, and 8a lymph nodal metastasis. The no. 6b correlations for lymph nodal metastasis were found with the no. 5 and no. 12a subgroups. The no. 6c correlations for lymph nodal metastasis were found with the no. 3, no. 4d, no. 5, no. 8a, and no. 9 lymph nodes.

**Table 3:** Correlation among the no. 6a, 6b, and 6c groups and other groups

Lymph node station	No. 6a (+)		No. 6b (+)		No. 6c (+)	
	*R* value* (95% CI)	*p* value	*R* value* (95% CI)	*p* value	*R* value* (95% CI)	*p* value
No. 1 (+)	− 0.01 (− 0.19 to 0.17)	0.897	− 0.01 (− 0.19 to 0.17)	0.897	0.02 (− 0.2 to 0.20)	0.827
No. 3 (+)	0.21 (0.03–0.38)	0.020	0.14 (− 0.04 to 0.31)	0.114	0.30 (0.13–0.46)	< 0.001
No. 4d (+)	0.14 (−0.04 to 0.31)	0.120	0.14 (− 0.04 to 0.31)	0.120	0.51 (0.36 to 0.63)	< 0.001
No. 4sb (+)	0.17 (− 0.01 to 0.34)	0.060	0.17 (−0.01 to 0.34)	0.060	0.04 (− 0.13 to 0.22)	0.617
No. 5 (+)	0.20 (0.03–0.37)	0.026	0.20 (0.03–0.37)	0.026	0.29 (0.11–0.45)	< 0.001
No. 6a (+)	–	–	0.20 (0.02–0.36)	0.030	0.02 (− 0.16 to 0.20)	0.804
No. 6b (+)	0.20 (0.02–0.36)	0.030	–	–	0.35 (0.18–0.49)	< 0.001
No. 6c (+)	0.02 (− 0.16 to 0.20)	0.804	0.35 (0.18–0.49)	< 0.001	–	–
No. 7 (+)	0.10 (− 0.08 to 0.27)	0.295	0.17 (−0.01 to 0.34)	0.067	0.19 (0.01 to 0.35)	0.038
No. 8a (+)	0.32 (0.15–0.47)	< 0.001	0.14 (− 0.04 to 0.31)	0.121	0.45 (0.30–0.58)	< 0.001
No. 9 (+)	0.12 (− 0.05 to 0.30)	0.177	0.12 (− 0.06 to 0.30)	0.177	0.38 (0.21–0.52)	< 0.001
No. 11p (+)	0.03 (− 0.14 to 0.21)	0.707	0.11 (− 0.06 to 0.29)	0.198	0.19 (0.02–0.36)	0.033
No. 12a (+)	0.03 (− 0.21 to 0.14)	0.707	0.23 (0.05–0.39)	0.012	− 0.07 (− 0.24 to 0.11)	0.459

*R value, the coefficient determined using the Spearman correlation test

### Risk factors for sub-pyloric (no. 6) lymph node metastasis

Risk factors for infra-pyloric lymph node metastasis were evaluated by the univariate and multivariate analysis. In the univariate analyses, the variables consisted of the clinicopathological features and the status of each lymph node station. Univariate analyses presented that metastasis to the no. 3, 4d, 5, 7, 8a, 9, and 11p stations and tumor location (lower third) were high risk factors of metastasis to infra-pyloric lymph nodes (*p* <  0.05) (Table 4). Finally, the multivariate analysis by logistic regression demonstrated that metastasis to no. 8a lymph nodes (OR = 1.329, 95% CI 1.055–1.674, *p* = 0.017) and no. 9 lymph nodes (OR = 1.250, 95% CI 1.036–1.509, *p* = 0.022) and lower third tumor of stomach (OR = 1.278, 95% CI 1.106–1.477, *p* = 0.001) were independent risk factors. Additionally, metastasis to no. 4d lymph nodes (OR = 1.193, 95% CI 0.988–1.425, *p* = 0.055) and poor histologic grade (OR = 1.173, 95% CI 0.980–1.405, *p* = 0.085) tended to be associated with sub-pyloric lymph node metastasis.

**Table 4:** Univariate and multivariate analysis of the risk factors for infra-pyloric (no. 6) lymph node metastasis

	Univariate analysis		Multivariate analysis*	
Risk factors	OR (95% CI)	*p* value	OR (95% CI)	*p* value
No. 1 (+)	1.015 (0.805–1.281)	0.898		
No. 3 (+)	1.334 (1.145–1.555)	< 0.001	1.035 (0.869–1.011)	0.670
No. 4sb (+)	1.055 (0.628–1.773)	0.840		
No. 4d (+)	1.666 (1.421–1.952)	< 0.001	1.193 (0.988–1.425)	0.055
No. 5 (+)	1.553 (1.232–1.959)	< 0.001	1.125 (0.931–1.384)	0.270
No. 7 (+)	1.240 (1.049–1.466)	0.013	0.977 (0.846–1.175)	0.975
No. 8a (+)	1.896 (1.584–2.269)	< 0.001	1.329 (1.055–1.674)	0.017
No. 9 (+)	1.566 (1.336–1.836)	< 0.001	1.250 (1.036–1.509)	0.022
No. 11p (+)	1.314 (1.080–1.599)	0.007	0.919 (0.754–1.120)	0.404
No. 12a (+)	1.249 (0.664–2.351)	0.491		
Age (≥ 65 years)	0.892 (0.748–1.065)	0.209		
Gender (female)	0.886 (0.747–1.051)	0.166		
Tumor location (lower third)	1.388 (1.189–1.619)	< 0.001	1.278 (1.106–1.477)	0.001
Tumor size (≥ 5 cm)	1.141 (0.972–1.339)	0.108		
Macroscopic type (type III-IV)	1.244 (1.048–1.475)	0.014	1.039 (0.892–1.211)	0.625
Histologic grade (≥ poor)	1.388 (1.189–1.619)	0.003	1.173 (0.980–1.405)	0.085
T stage (T4)	1.168 (0.992–1.374)	0.064	0.997 (0.843–1.179)	0.973
N stage (N2–3)*	1.514 (1.295–1.769)	< 0.001		

*Not entered into the regression model due to the potentially confounding effect on lymph node status

### Survival outcome analyses

**Fig. 1:**
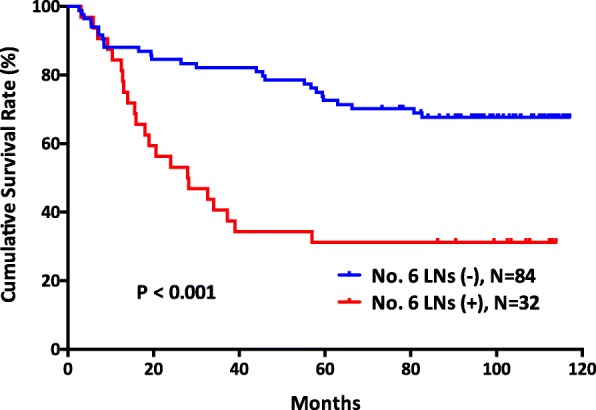
The survival outcomes of gastric cancer patients between those with negative no. 6 lymph nodes and positive no. 6 lymph nodes (*p* < 0.001)

**Fig. 2:**
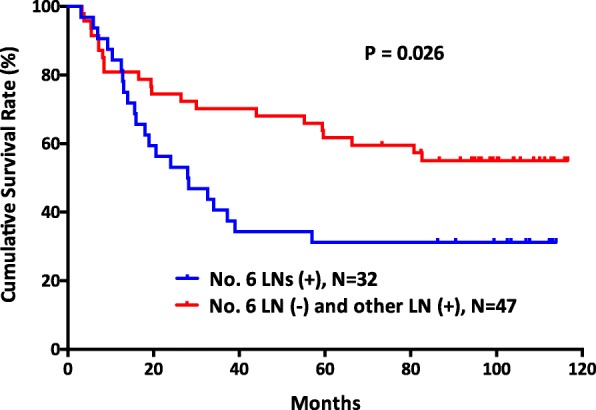
The survival outcomes of gastric cancer patients between those with metastasis in no. 6 lymph node station and other lymph node stations (*p* = 0.026)

**Fig. 3:**
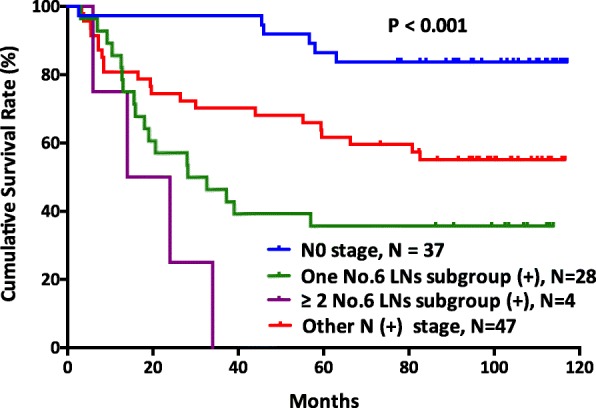
The survival curves of gastric cancer patients among those with N0 stage, metastasis in one subgroup of no. 6 lymph nodes, metastasis in more than two subgroups of no. 6 lymph nodes, and metastasis in other lymph node stations (*p* <  0.001)

There was a significant survival difference between the no. 6 node-positive groups and the no. 6 node-negative groups (*p* <  0.001) (Fig. 1). In addition, patients with no. 6 lymph nodal metastasis had significantly poor survival outcomes compared with those patients with other lymph nodal metastasis (*p* = 0.026) (Fig. 2). Furthermore, patients with more than two no. 6 sub-station metastasis had worse survival outcomes than patients with other lymph node station metastasis, patients with one no. 6 sub-station metastasis, and patients with N0 stage (*p* <  0.001) (Fig. 3).

In univariate survival analysis (Table 5), tumor size (≥ 5 cm), macroscopic type (type III-IV), histologic grade (≥ poor), T stage (T4), and N stage (N1-3) were prognostic risk factors. And in the multivariate analysis (Table 5), tumor size (≥ 5 cm) and N stage (N1-3) were independent prognostic risk factors.

**Table 5:** Univariate and multivariate survival analysis patients included in this study

	Univariate analysis		Multivariate analysis	
Risk factors	HR (95% CI)	*p* value	HR (95% CI)	*p* value
Age (≥ 65 years)	0.759 (0.388–1.486)	0.421		
Gender (female)	0.919 (0.500–1.686)	0.784		
Tumor location (lower third)	1.918 (1.030–3.569)	0.039	1.228 (0.637–2.367)	0.539
Tumor size (≥ 5 cm)	4.428 (2.373–8.261)	< 0.001	3.095 (1.573–6.089)	0.001
Macroscopic type (type III-IV)	2.809 (1.592–4.956)	< 0.001	1.441 (0.787–2.636)	0.236
Histologic grade (≥ poor)	3.400 (1.057–10.940)	0.040	2.586 (0.757–8.835)	0.129
T stage (T4)	3.408 (1.698–6.841)	< 0.001	1.324 (0.609–2.877)	0.478
N stage (N1-3)	4.633 (1.968–10.900)	< 0.001	2.608 (1.046–6.506)	0.039
Chemotherapy (without)	1.317 (0.747–2.321)	0.341		

## Discussion

Lymphadenectomy is fundamental to gastric cancer surgery, and a systematically performed lymphadenectomy not only aims to excise the lymph nodes but also to achieve en bloc tissue dissection along the lymphatic drainage area. Theoretically, complete resection of gastric cancer cells with metastatic potential can extend the survival duration of patients. However, during the operation, surgeons can only manage visible and suspicious lesions. Therefore, prophylactic resection, such as radical D2 lymphadenectomy, is highly important for gastric cancer patients. Over recent decades, D2 lymphadenectomy has been recognized as the standard surgical treatment strategy for advanced gastric cancers [4]. According to the Japanese Gastric Cancer Treatment Guideline, the no. 6 lymph nodes, which are located along the first branch and proximal part of the REGA to the confluence of the REGV and the ASPDV, must be completely resected during D2 distal gastrectomy and D2 total gastrectomy [5, 6]. In this study, we found that the metastasis rate was 28.1% of no. 6 lymph nodes, and all of its three subgroups had the potential for involvement by metastatic cancer cells. In addition, the patients with metastasis to the no. 6 lymph nodes had significantly poorer survival outcomes (*p* <  0.001). Therefore, this study did not aim to advocate subgrouping of the infra-pyloric lymph nodes during the operation or to compare the subgroup methods with those of the Japanese report [15, 16]. However, we primarily aim to emphasize complete and thorough resection of the infra-pyloric lymph nodes during standard D2 gastric cancer surgery.

Special anatomical features of the infra-pyloric area exist, such as the REGV is not completely concomitant with the REGA. In addition, anatomical variations of another artery, the infra-pyloric artery (IPA), also exist. According to the origin of the IPA, the Japanese investigators divided these variations into three subtypes: distal variations (from the anterior superior pancreatoduodenal artery, 64.2%), caudal variations (from the REGA, 23.1%), and proximal variations (from the gastroduodenal artery, 12.7%) [16]. Therefore, due to the intricate network of blood vessels, complete lymphadenectomy is technically complicated for the infra-pyloric region. Moreover, this region directly abuts the pancreas and transverse mesocolon, and thus, the potential risk of injury to these structures exists. Therefore, all these factors will increase the operative difficulties and risk of postoperative complications.

To achieve a complete and safe resection of the infra-pyloric lymph nodes, developing an appropriate surgical plane is particularly important. Nevertheless, there is a lack of high-grade evidence that proves that bursectomy can improve the prognosis of gastric cancer patients. However, Blouhos et al. believe that by the surgical plane of right-sided bursectomy, the infra-pyloric lymph nodes can easily attain en bloc resection [21]. Our experience is that a right-sided bursectomy approach can facilitate the development of the correct surgical plane and facilitate lymphadenectomy in this area. First, identify the space between the anterior and posterior regions of the transverse mesocolon to the lower edge of the pancreas; second, upward to surface of pancreas, clear the adipose tissue and lymph nodes from the surface of pancreatic head; finally, expose the root of REGV, REGA, and IPA in sequence, and then, the infra-pyloric lymph nodes can easily and completely remove. In addition, cancer cells may be shed into the peritoneal cavity during lymph node dissection when surgeons transect the lymphatic vessels [22]. A previous study reported that the inappropriate closure of lymphatic vessels could lead to increased carcinoembryonic antigen mRNA levels and the release of free gastric cancer cells in an ex vivo model [23]. Therefore, to achieve oncological complete lymph nodal resection, proper dissection technique and the correct surgical plane are important to minimize potential free gastric cancer cell shedding during the operation.

The reported metastasis rate in this group is from 19.2 to 42.9% [12–14]. In our previous study, we reported that metastasis to no. 6 lymph nodes is significantly correlated with positive no. 8a lymph nodes and depth of tumor invasion [18]. In the present study, except for the no. 8a lymph node station, no. 9 lymph nodal positivity and tumor location were also risk factors for no. 6 lymph nodal positivity. The index of estimated benefit from lymph node dissection (IEBLD), which is used to evaluate the therapeutic value of lymphadectomy, reported that the metastasis rate was 19.2% and the IEBLD was 11.6 for the no. 6 nodes in gastric cancer patients [12]. Generally, Chinese patients had more advanced tumor stages than Korean and Japanese patients [24]. In our study, we also found a higher rate of metastasis to the no. 6 lymph nodes than that reported by Imamura et al. [12]. In the survival analysis of the present study, patients with positive no. 6 lymph nodes had poorer survival outcomes than those with negative no. 6 lymph nodes. Therefore, complete infra-pyloric lymphadenectomy is crucial. Importantly, in the present study, we wish to emphasize the importance of complete resection of the no. 6 lymph nodes rather than conclude that the no. 6 lymph node station is more important than any other lymph node station. We believe that all lymph node stations requiring removal for D2 lymphadenectomy are equally important and should be treated seriously.

This study has a few limitations. The sample size and the nature of a retrospective study are its major limitations. Because of the limitation of sample size, this study did not compare the survival outcomes between patients with positive and negative status for each subgroup of infra-pyloric lymph node stations by different tumor stages. Also, due to the limitation of sample size, the results of the correlation analysis are weak, and the relationship of the no. 6 lymph nodes with other parameters cannot be determined. In addition, due to the limitations of a retrospective study, we did not include comprehensive clinicopathological characteristics in the statistical analysis, such as Lauren’s classification, lymphovascular invasion, perineural invasion, and other parameters.

## Conclusions

In conclusion, metastasis to the no. 6 lymph nodes is related to the survival outcomes of gastric cancer patients. Complete resection of the no. 6 lymph nodes is important for patients who undergo gastric cancer surgery.

## Abbreviations

AJCC: American Joint Committee on Cancer
ASPDV: Anterior superior pancreaticoduodenal vein
CI: Confidence interval
IEBLD: Index of estimated benefit from lymph node dissection
IPA: Inferior pylorus artery
OR: Odds ratio
REGA: Right gastroepiploic artery
REGV: Right gastroepiploic vein
